# Multifocal *Serratia Marcescens* infection in a healthy adult

**DOI:** 10.1016/j.jdcr.2022.10.006

**Published:** 2022-10-13

**Authors:** Francesca Genera, Faraz Yousefian, Gerard Danosos, Suzanne Friedler, Victor Martinez

**Affiliations:** a University of the Incarnate Word School of Osteopathic Medicine, San Antonio, Texas; b Center for Clinical and Cosmetic Research, Aventura, Florida; c Department of Dermatology, St. John's Episcopal Hospital, New York, New York

**Keywords:** granulomatous necrosis, infection, *Serratia Marcescens*, CGD, chronic granulomatous disease

## Introduction

*Serratia marcescens* is a gram-negative bacillus belonging to the Enterobacteriaceae family and is commonly associated with respiratory tract infections, urinary tract infections, and endocarditis.^1^ On extremely rare occasions, this facultative anaerobe has been associated with primary cutaneous infections.^2^ These infections tend to manifest in immunocompromised individuals or those with an underlying medical condition, such as alcoholic cirrhosis, venous insufficiency, or chronic granulomatous disease (CGD).^1^ Acutely, *S* *marcescens* causes cellulitis in combination with skin abscess formation.^3^ In chronic infections, it typically causes necrotic nodules and ulcers, although a rare case of granulomatous inflammation has been reported.^3^ Literature about the incidence of primary cutaneous Serratia infections is extremely limited. Here, we report a case of a healthy 21-year-old Asian-American woman with a 3-week history of rash and ulceration as a result of *S* *marcescens*.

## Case report

A 21-year-old Asian-American woman presented to the outpatient dermatology clinic with painless and nonpruritic erythematous papules and plaques on her arms and back over the past 3 weeks. These ulcers initially developed on her forearms and eventually progressed to back. She denied itchiness or fever before the rash onset. No inciting event was identified. She reported a history of childhood chickenpox and keloids. Her family history was noncontributory. She denied a smoking history and was not on any medication. A pertinent review of the systems was negative for fever, pruritus, dysuria, or shortness of breath. She reported seasonal allergies and an allergy to peanuts; however, she denied allergen exposure at the time of presentation. She had no history of dermatologic disease. Four months prior, she had traveled to the Philippines.

**Fig 1:**
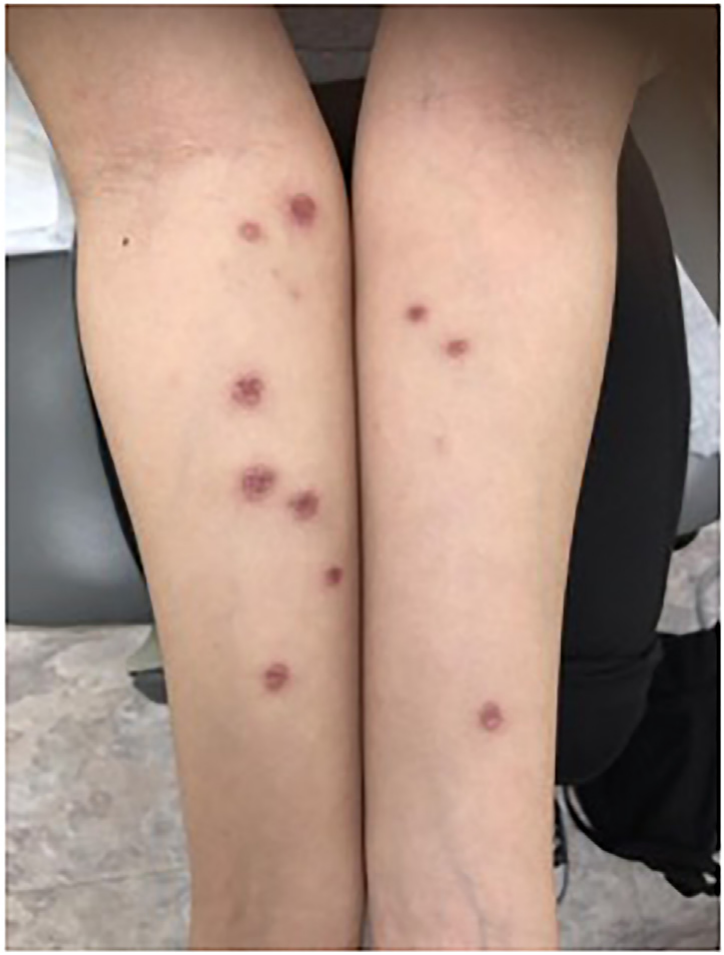
Erythematous, indurated, necrotic plaques and papules on the forearms prior to therapy.

**Fig 2:**
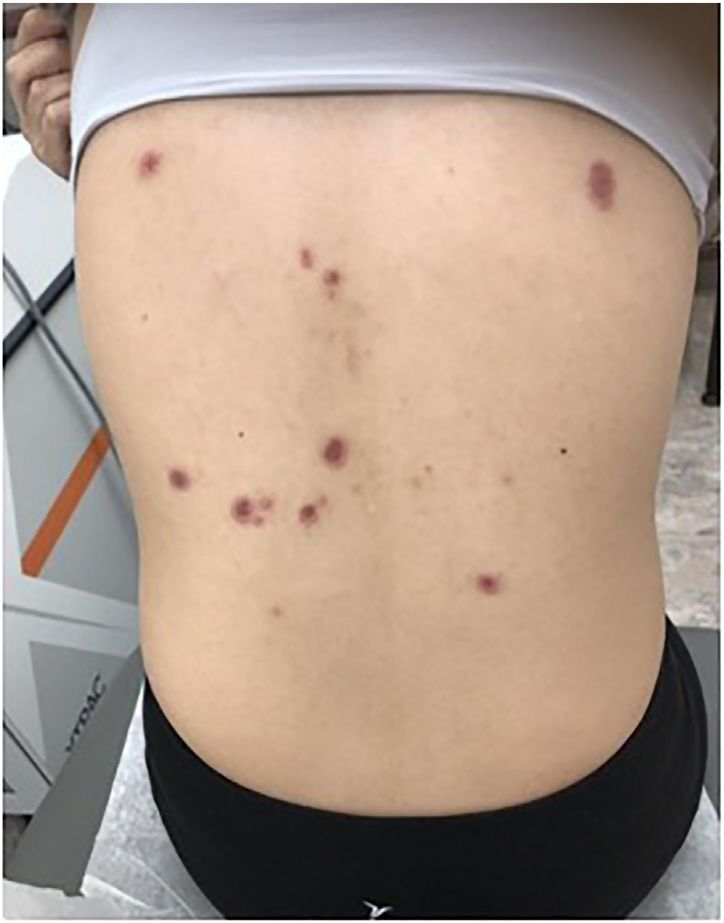
Erythematous, indurated, necrotic plaques and papules on the back prior to therapy.

On physical examination, erythematous to violaceous ovoid to round circumscribed necrotic papules and plaques were present on the right forearm and back (Figs 1 and 2). Liver function tests, thyroid function tests, and complete blood cell count were within normal limits. QuantiFERON gold, HIV, and rapid plasma reagin were negative. Results of autoimmune antibody assays, including antinuclear antibodies, revealed no abnormalities. Based on the patient’s stable vital signs, review of systems, physical examination findings, and normal complete blood cell count results, a blood culture for bacteremia was not indicated.

**Fig 3:**
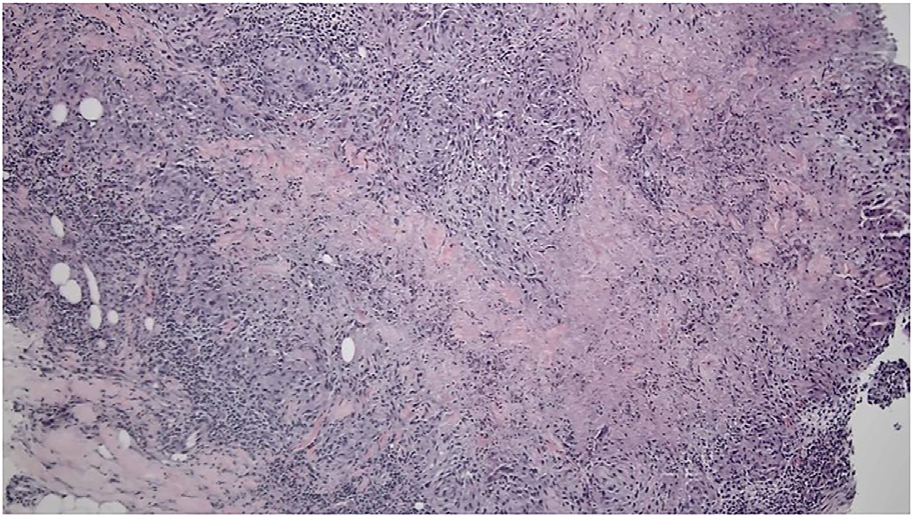
Microscopic sample demonstrating collections of epithelioid histiocytes with focal necrosis.

Punch biopsies and tissue cultures were taken from multiple locations for histologic evaluation to ensure adequate sampling, including one at the upper portion of the right arm, the middle portion of the right arm, lateral aspect of the right arm, and left side of the back. The histologic study of biopsy specimens revealed a dense collection of neutrophils and histiocytes, confirming granulomatous dermatitis with necrosis. Ziehl–Neelsen stain (BIN1) and fungal cultures were negative. Tissue culture from 2 different samples revealed heavy growth of *S* *marcescens*; the second culture was obtained during the follow-up visit to confirm infection with *S* *marcescens* with sensitivity to fluoroquinolones and aminoglycosides. Within the dermis, there were collections of epithelioid histiocytes with focal necrosis (Fig 3).

Differential diagnoses considered for the patient included infectious causes, such as atypical *Mycobacterium*, deep fungal infections, such as *Blastomycosis* or *Cryptococcus*, or neutrophilic dermatoses, all of which were ruled out with tissue cultures and hematoxylin and eosin findings. Once *S* *marcescens* was identified, the patient was started on ciprofloxacin (500 mg, 1 tablet, orally twice a day for 3 weeks, 21 days orally twice a day). At the next follow-up, the patient showed mild clinical improvement with persistent ulcers and reported insomnia; subsequently, she was switched to a 4-week course of levofloxacin orally. The next follow-up was conducted through telemedicine because the patient was traveling; she reported significant improvement and resolution of cutaneous ulcers.

## Discussion

Erythematous cutaneous ulcers have been linked to a variety of causes, with the most prevalent being bacterial infections by Staphylococcal and Streptococcal species. In contrast, *S* *marcescens*, a gram-negative, motile bacillus belonging to the Enterobacteriaceae family, rarely infects the human skin, and seldom in young, healthy individuals. *S* *marcescens* has been linked to cutaneous infections on a very limited number of reported occasions. Necrotizing fasciitis, cellulitis, and dermal abscesses are the most frequent presentations among these reports.^1^^,^^4^ Eight cases of skin ulceration in predominantly immunocompromised individuals caused by *S* *marcescens* have been reported in the literature.^1^ However, there have not been any studies that have identified *S* *marcescens* as a primary cause of painless, erythematous, indurated plaques and papules.

*S marcescens* has been identified as opportunistic in nature because this organism does not routinely cause primary invasive disease. A systematic review conducted by Veraldi and Nazzaro^1^ identified risk factors for skin ulcers caused by *S* *marcescens,* including chronic venous insufficiency, alcoholic-induced hepatic cirrhosis, obesity, preexisting leg ulcers, CGD, and trauma. There was only 1 case of *S* *marcescens* in an immunocompetent individual reported. Six of the 8 cutaneous *S* *marcescens* ulcers reported by Veraldi and Nazzaro^1^ were in men, with an average age of 52 and the youngest being 18 years old. Seven of the patients had leg ulcers, whereas 1 patient with CGD presented with ulcers of the arm, thigh, and scrotum.

In a study by Carlesimo et al^5^ a young immunocompetent man with no preexisting risk factors presented with painful leg ulcers because of *S* *Marcescens* infection. The authors suggested that the patient may have had an underlying genetic predisposition for the primary cutaneous disease. We present a similar case of a 21-year-old immunocompetent woman with recent travel to Asia, presenting with painless, erythematous indurated plaques and papules of the upper extremities and back. Multiple biopsies of the ulcers were taken for tissue culture and histology on several visits and identified *S* *marcescens* as the culprit. The patient underwent further workup to rule out immune deficiencies and autoimmune conditions, which returned negative results. Following treatment with levofloxacin, the cutaneous infection improved significantly with the resolution of the ulcers. Our case suggests that *S* *marcescens* may be pathogenic and an underlying cause of primary cutaneous ulceration. Further research into genetic testing could offer insight into the cause of *S* *marcescens* skin infections in immunocompetent people.

## Conflicts of interest

None disclosed.
